# The regulation of transcription elongation in embryonic stem cells

**DOI:** 10.3389/fcell.2023.1145611

**Published:** 2023-02-16

**Authors:** Xuepeng Wang, Yudan Fan, Qiang Wu

**Affiliations:** The State Key Laboratory of Quality Research in Chinese Medicine, Macau University of Science and Technology, Taipa, Macao SAR, China

**Keywords:** embryonic stem cells (ESCs), transcription elongation, RNA pol II, P-TEFb, alternative splicing, promoter-proximal pausing

## Abstract

Transcription elongation is a fundamental molecular process which is accurately regulated to ensure proper gene expression in cellular activities whereas its malfunction is associated with impaired cellular functions. Embryonic stem cells (ESCs) have significant value in regenerative medicine due to their self-renewal ability and their potential to differentiate to almost all types of cells. Therefore, dissection of the exact regulatory mechanism of transcription elongation in ESCs is crucial for both basic research and their clinical applications. In this review, we discuss the current understanding on the regulatory mechanisms of transcription elongation mediated by transcription factors and epigenetic modifications in ESCs.

## 1 Introduction

The first step of gene expression is transcription which is a process that can produce the RNA according to the DNA template allowing passage the genetic information from DNA to RNA by the transcription machine. Transcription is executed by RNA polymerase (Pol II) and its associated factors and can be divided into transcription initiation, transcription elongation and transcription termination according to the different stages. Briefly, transcription initiation contains the formation of pre-initiation complex (PIC), initiation of transcription, and pausing of Pol II after it transcribes 25–50 nucleotides (promoter-proximal pausing Pol II). Transcription elongation is a critical process that releases pausing Pol II on proximal promoter and overcomes nucleosome barriers to produce nascent RNA. To enter the transcription elongation phase, Pol II escapes from the pause for productive elongation which needs positive elongation factor b (p-TEFb) and other supporting auxiliary factors. In addition, owing to the coupling between the elongation and RNA processing including nascent RNA 5’ capping and alternative splicing (AS), elongation rate can affect the splicing efficiency (Luco et al., 2011). The transcription termination mainly consists of the release of nascent RNA and recycling of Pol II.

At the transcription initiation stage, Pol II and auxiliary factors need to be recruited to gene promoter regions. The phenomena of poised RNA polymerase II at the proximal promoter regions without productive elongation was found at the *Hsp70* gene in *Drosophila* and at the *c-Myc* and *c-Fos* genes in mammalian cells (Kwak and Lis, 2013; Chen et al., 2018). In fact, the presence of promoter-proximal paused Pol II is common and conserved since 40%–46% of active genes possess paused Pol II in mammals while 70%–89% of active promoters contain paused Pol II in *Drosophila* embryonic cell lines (Core et al., 2008; Min et al., 2011; Core et al., 2014). In mouse ESCs, the core pluripotency transcription factors such as Oct4 and Nanog are highly expressed, yet possess paused Pol II at proximal promoter regions (Min et al., 2011). Furthermore, the modulation of transcription elongation is highly integrated by generally associated elongation factors and context-dependent factors in different cell types. For example, in ESCs, BRD4 recruits mediator proteins and CDK9 of p-TEFb catalysis subunit to Super-enhancer (SE)-associated pluripotency genes to maintain ESC identity (Di Micco et al., 2014). Similarly, c-Myc binds to p-TEFb to control the transcription elongation of its target genes in pluripotent stem cells (Rahl et al., 2010). In immune cells, NF-kB can interact with cyclin T1 of p-TEFb subunit at NF-kB target genes when responding to the stimulation of TNF-α (Barboric et al., 2001).

ESCs which are derived from the inner cell mass (ICM) possess unique features with self-renewal and pluripotency. The unique features of ESCs are maintained by complex mechanisms which are comprised of the cooperation of multiple transcription factors, key signaling pathways, and epigenetic modifications to ensure the pluripotent genes to be properly expressed while the lineage genes to silenced (Wang and Wu, 2022). Previous studies have comprehensively discovered the gene regulatory mechanisms in both maintenance and acquisition of pluripotency. For example, core transcription factors OCT4/SOX2/NANOG not only regulate many downstream genes but also their own genes to maintain pluripotency in ESCs. However. Most of these studies primarily focus on how regulatory signals and key transcription factors contribute to gene expression at the level of the transcription initiation and recruitment of RNA Pol II to gene promoters. Recently, increasing evidence showed that gene expression is also largely regulated at the level of transcription elongation stage during which the release of promoter-proximal pausing Pol II and the production of full-length transcript efficiently are involved (Saunders et al., 2006). Interestingly, transcription elongation seems to be a switch between pluripotency maintenance and differentiation in ESCs. For example, in plants, activating transcriptional elongation by nuclear accumulation of IYO initiates differentiation in *Arabidopsis* whereas overexpression of IYO accelerates the onset of differentiation (Sanmartin et al., 2012). Conversely, reducing the expression of IYO leads to differentiation being delayed in the plant. Furthermore, maintenance of vertebrate precursor germ cells involves the global suppression of transcriptional elongation (Kumano et al., 2011). Furthermore, Polycomb group proteins (PcGs) are enriched on many developmental regulators which are related to stalled Pol II, and depletion of Polycomb subunits Ring1A/B releases paused Pol II and triggers differentiation in ESCs (Stock et al., 2007).

Given that ESCs have unique gene expression profile and dynamic chromatin structure, dissecting the regulatory mechanism of transcription elongation in ESCs is fundamental for both basic research and clinical applications. Recent cutting-edge technologies including high-resolution microscopy and high-throughput sequencing have provided much more comprehensive data which can facilitate the research on transcription elongation mechanisms. In this review, we summarize the key events in transcription from the establishment of promoter-proximal pausing Pol II to productive elongation. We focus on the recent advances in molecular mechanisms of transcription elongation regulation by transcription factors and epigenetic modifications in ESCs.

## 2 The key events of the transcription cycle in eukaryotes

Gene transcription is to passage the genetic information stored in DNA sequence to messenger RNAs in both prokaryotes and eukaryotes. Three differences make the transcription more complex in eukaryotes (Madan Babu and Teichmann, 2003; Nilkanta and Bagchi, 2018), 1) Introns that do not code proteins, thus splicing is needed; 2) Nucleosomes in eukaryotes that consist of octamer histones and wrapped with 147 bp DNA, are major barriers for transcription; 3) Enhancers and super-enhancers which can speed up transcription to rapidly respond on specific signals and stimulation of environments. Consistently, the regulatory mechanisms of transcription are dependent on different cellular conditions and specific target genes.

### 2.1 From transcription initiation to promoter-proximal pausing Pol II

**FIGURE 1:**
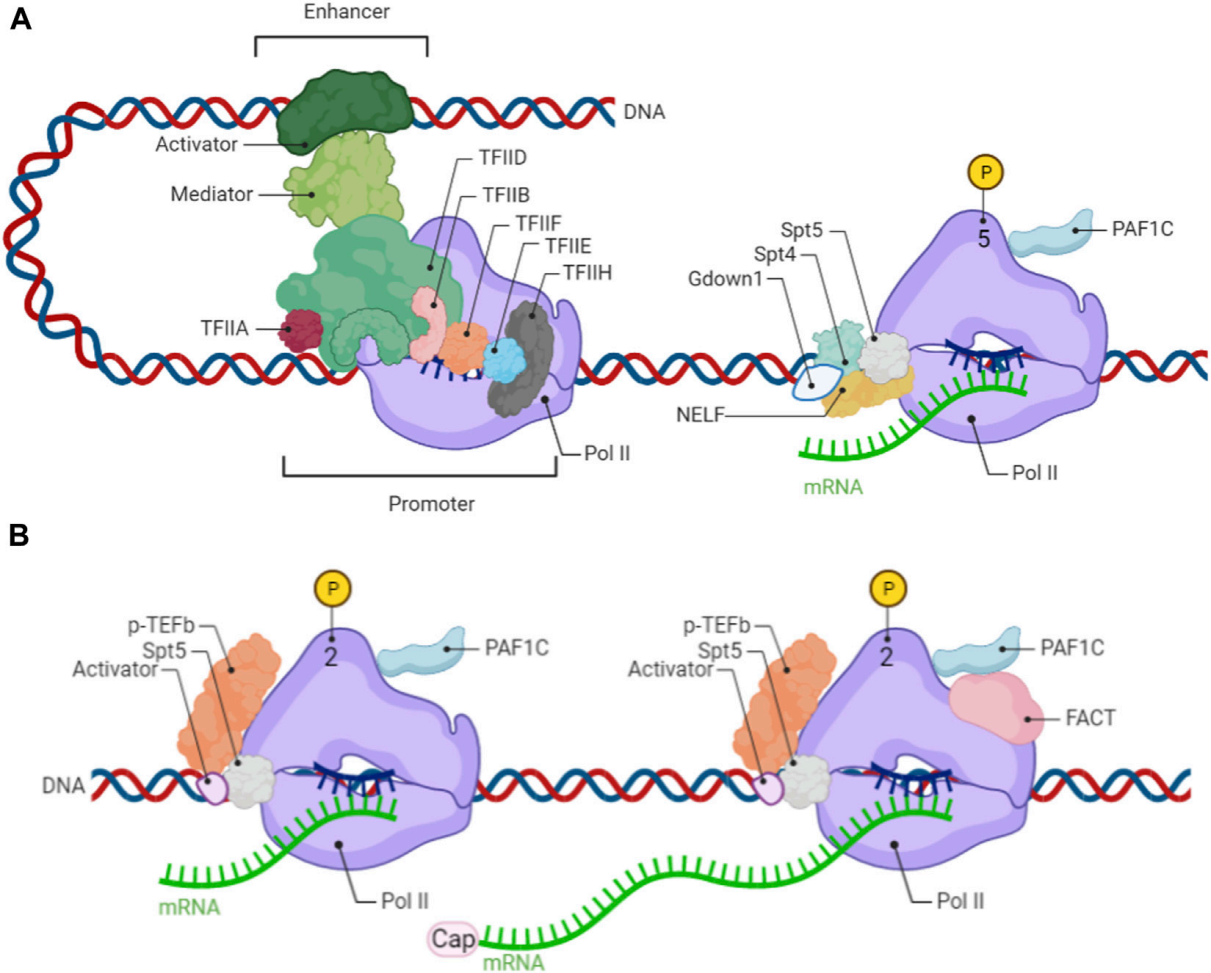
**(A)** From transcription initiation to pausing Pol II on proximal promoter **(B)** Release of pausing Pol II on proximal promoter to productive elongation.

Transcription stars from promoter regions of genes that are generally enriched with cis-regulatory elements, such as TATA box, which can be recognized by the general transcription factor (GTFs) TFIIA/B/D/E/F/H. For gene activation, general transcription factors bind to cis-acting element at promoter regions and then recruit Pol II to form pre-initiation complex (PIC). Transcription initiation refers to that Pol II produces nascent RNA along the template DNA after the helicase unwind DNA (Figure 1). During transcription initiation, there is abortive transcription in which the transcription terminates prematurely and the nascent RNA is shorter than 10 bp, thus functioning as a checkpoint of promoter control. Further extend nascent RNA from 20–60 bp, the peaks of promoter-proximal pausing Pol II measured by ChIP-seq on about half of active genes in mammals have been observed. During this process, general transcription factors such as TFIIB/F disassociate from the Pol II complex while the carboxy-terminal domain (CTD) extended from the core of the Pol II is phosphorylated at Ser5 (Pol II Ser5P) by CDK7 of TFIIH and Mediator. Phosphorylated Pol II then recruits SPT4/5, known as DSIF (DRB Sensitivity-Inducing Factor) following the negative elongation factor (NELF) recruitment, leading to Pol II pausing at promoter-proximal regions. In addition to DSIF and NELF supporting paused Pol II, Gdown1 and PAFC1 (Pol II-associated factor complex 1) also participate in this process. Gdown1 can compete with TFIIF and inhibit TTF2 (transcription termination factor 2) to stabilize the pausing Pol II. Interestingly, PAFC1 has dual functions according to its conformation, functioning as a pausing factor when binding to the form of phosphorylation Pol II at Ser5, or as an elongation factor when binding to the form of phosphorylation Pol II at Ser2. Overall, promoter-proximal pausing Pol II can maintain open chromatin accessibility and facilitate rapid gene expression when responding to signals and stimulation in environments.

### 2.2 Transitions from the pausing Pol II on promoter-proximal to productive elongation

#### 2.2.1 Release of pausing Pol II on the proximal promoter

**FIGURE 2:**
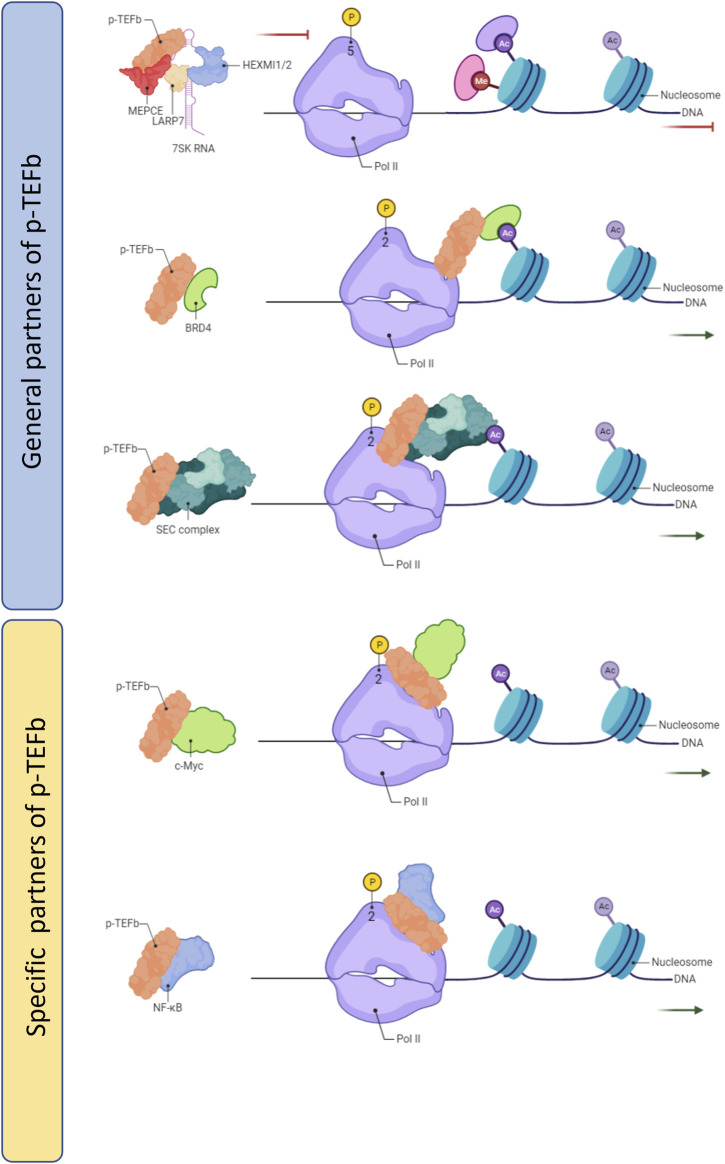
The partners of p-TEFb.

Release of pausing Pol II on the proximal promoter regions into productive elongation is a rate-limitation step in gene expression and is highly regulated by multiple factors. The transition from pausing Pol II to release is mainly driven by the positive transcription p-TEFb complex, comprising cyclin T1 and cyclin-dependent kinase 9 (CDK9). There are serval general partners and cellular-specific partners of p-TEFb which can make it into an inactive or active state (Figure 2). 7SK snRNP is a common interactor of p-TEFb and inactivates it until released (Guo and Price, 2013). Two other general partners of p-TEFb are BRD4 and super elongation complex (SEC) which can recruit active p-TEFb to promoter-proximal pausing Pol II to enter the productive elongation stage. Both BRD4 and SEC can recognize acetylated histones at promoter or enhancer regions thereby recruiting p-TEF-b (Zhou and Yik, 2006; Lin et al., 2011). For the context-depend on partners of p-TEFb including NF-kB in many types of cancer cells (Barboric et al., 2001), SOX2 and SOX10 in Schwann cells (Arter and Wegner, 2015), Mediator both in serval cancer cells and pluripotent stem cells (Donner et al., 2010; Li et al., 2019), and c-MYC in pluripotent stem cells (Rahl et al., 2010). After p-TEFb interacts with pausing Pol II, CDK9 of p-TEFb directly phosphate Pol II at ser 2, NELF and DSIF (Spt4/5). The phosphorylation of NELF, Spt4 and Gdown1 dissolve from the Pol II complex, while Spt5 changes its function toward a positive elongation factor. Owing to phosphorylation Pol II at Ser 2 by p-TEFb, PAF1C changes its conformation and functions as a positive elongation factor. Moreover, SPT6 (Suppressor of Ty 6) exerts an important function in releasing Pol II through PAF1C recruitment and NELF removal on the surface of RNA Pol II (Aoi et al., 2022). These series of changes can release the promoter-proximal paused Pol II so that transcription elongation proceeds.

#### 2.2.2 Overcome nucleosome barrier during the effective elongation

After pause release, the elongating Pol II still needs to overcome the nucleosome barrier to smoothly produce RNA transcript. Chromatin remodelers and histone disassembly and reassembly can mediate the process. FACT (facilitates chromatin transcription), consists of SPT16 and SSRP1 subunits, participates in the displacement of H2A–H2B dimers of nucleosomes to facilitate Pol II transcribing. SPT6, a histone chaperone, can directly interact with histones H3/H4 to facilitate Pol II transcribing. PAF complex also can relieve the nucleosome barrier during elongation and seems to serve as a platform to recruit elongation-related factors, such as SEC (He et al., 2011), FACT (Pavri et al., 2006). In addition, activation of PARP (poly ADP-ribose polymerase) can form poly (ADP-ribose) chains and then cause rapid nucleosome loss before elongation of Pol II through the gene body (Petesch and Lis, 2012).

The elongation rate is crucial for transcription regulation and is related to the level and processing of transcripts, such as alternative splicing. There are a set of factors that can enhance the elongation rate. Both eleven-nineteen lysine-rich in leukemia (ELL) and elongin can increase the elongation rate by suppressing transient pausing of Pol II and enhancing Pol II through the gene body (Smith et al., 2008; Zhou et al., 2009). In addition, TFIIF prompts the elongation rate by promoter escape, in line with its favorite promoter locations (Jonkers and Lis, 2015; Core and Adelman, 2019).

## 3 Dysfunction of components of transcription elongation causes varied phenomena in ESCs

ESCs can rapidly respond to developmental stimulation to either differentiate into specific types of cells or maintain the pluripotent state. Modulations of transcription elongation are important for the balance of pluripotency maintenance and induced differentiation (Efroni et al., 2008). Therefore, dysfunction of different transcription elongation factors can cause various phenotypes in ESCs.

### 3.1 Transcription elongation rate affects ESC differentiation and early embryo development

The rate of transcription elongation is associated with the timing and output of mRNA production and is influenced by a positive effect of histone H3K79me2 and negative effects of exon density and CG content (Jonkers et al., 2014). It varies in different genes in specific cell types under different conditions, and is highly related to the determination of alternative splicing (AS) patterns including exon skipping and inclusion (Dujardin et al., 2014; Herzel et al., 2017; Aslanzadeh et al., 2018; Maslon et al., 2019). AS is a major contributor to diversity of RNA and protein isoforms and highly regulated by transcription elongation rate and splicing factors (Pandya-Jones and Black, 2009). During transcription elongation, Pol II produces the nascent RNA with alternation of exons and introns. Then splicing factors are recruited to process alternative splicing. Usually, faster transcription elongation rate causes exon skipping due to lack of time for splicing factor to recognize it, while slower rate allows splicing machinery to recognize and splice out exons, leading to exon inclusion. Though a study showed that slow elongation can either up or downregulates exon inclusion depending on the exon (Dujardin et al., 2014), this “cotranscriptional” feature allows quicker transcriptional responses to stimuli and disruption of the elongation rate significantly affects the development and AS. Indeed, in ESCs, reduction of the global transcription elongation rate by knocking in a RNA Pol II mutant (C4/R749H) results in clear changes in AS, early embryonic lethality and defects on neural lineage by (Maslon et al., 2019).

### 3.2 Loss of transcription elongation factors impairs the identity of ESCs

Elongation factors are a set of proteins to facilitate transcription elongation, some of which are highly expressed in ESCs but not in somatic cells, suggesting that they may participate in the maintenance of ESCs. Transcription elongation factor TFIIS (also named as SII) can prevent paused polymerase backtracking and facilitate Pol II to create a new RNA 3′-OH in the active site (Cheung and Cramer, 2011). TFIIS is coded by Tcea1, Tcea2, and Tcea3. Among those, depletion of Tcea1, which is the original form, referred to as ‘‘general SII,’’ causes mice to die of severe anemia at mid-gestation stage (Ito et al., 2006). While disrupting Tcea3 expression affects the mouse ESC differentiation and embryo development by prompting the Lefty1-Nodal-Smad2 pathway.

RNA polymerase II-associated factor 1 (Paf1) and its homolog PD2 are involved in the regulation of promoter-proximal pausing Pol II and further transcriptional elongation. Paf1/PD2 are highly expressed in ESCs and involved in pluripotency maintenance *via* interacting with Oct4 and Pol II to regulate Oct4-mediated gene expression (Ponnusamy et al., 2009). Depletion of Paf1/PD2 causes the loss of ESC identity and decreased core pluripotent markers Oct4, Sox2, Nanog, and Shh and increased endodermal differentiation markers Gata4, Gata6, and Fgf8. Interestingly, PHD finger protein 5a (Phf5a) can directly interact with Paf1 complex (Paf1C) and control its integrity, thus facilitate the expression of pluripotency genes through Paf1C binding in ESCs (Strikoudis et al., 2016). In addition, Paf1C is involved in the deposition of histone modifications which are associated with transcription elongation. Paf1C maintains the level of histone H3K79me and suppresses the deposition of H2BK120-ub in ESCs. Depletion of Phf5a can increase the level of H2BK120-ub modification while decreasing the level of H3K79me modification at core pluripotent genes including *Nanog*, *Pou5f1*, and *Sox2* (Strikoudis et al., 2017).

### 3.3 Transcription elongation factors participate in totipotency maintenance

Blastomeres from zygote to two-cell-stage (in mice) or to 8-cell-stage blastomeres (in human) are normally considered totipotent as they have potential to give rise to both embryonic and supportive extra-embryonic tissues (Wang and Wu, 2022). In the early embryo, the first transcription event is zygotic genome activation (ZGA) which divides into minor ZGA (generating a small set of coding transcripts as well as low levels of non-productive transcripts through promiscuous transcription) at one-cell stage and major ZGA (during which thousands of genes start to be transcribed) in the at the late two-cell stage (Abe et al., 2015; Liu et al., 2020). Interestingly, transcription elongation also participates in the maintenance of totipotency. The transient inhibition of minor ZGA using a reversible inhibitor of Pol II elongation can prevent most embryos from developing beyond the two-cell stage, while CDK9 and SPT5 are major ZGA regulators (Abe et al., 2022).

Totipotent two-cell-like cells (2CLCs) share features with the two-cell-stage mouse embryos in transcriptome, epigenome, and developmental potential (Genet and Torres-Padilla, 2020). Negative elongation factor A (NELFA) promotes a 2CLCs fate by interacting with Top2a to drive the key 2CLCs regulator Dux expression. (Hu et al., 2020). In addition, depleting NELF-B from the zygote to the 2-cell stage results in progressive developmental arrest from the 4-cell stage and prevents blastocyst formation. However, NELF-C/D and NELF-E are not necessary for global transcription in 2-cell-stage embryos. Taken together, these suggest that the non-redundant function of the NELF complex in the early embryo development and totipotency maintenance (Abuhashem et al., 2022).

## 4 Transcription elongation is highly regulated in ESCs

### 4.1 Epigenetic regulation of transcription elongation in ESCs

The chromatin of ESCs is generally more open and more dynamic with distinct bi-valent histone modifications (co-harboring repressive histone H3K27me3 mark and active histone H3K4me3 mark at many developmental genes). Epigenetic modifications including histone modifications and DNA methylation are mainly determined by multiple epigenetic modifiers. These epigenetic modifiers (writers/erasers) introduce/remove co-valent modifications on DNA and histones by their catalysis activity. The reader proteins can recognize these epigenetic modifications through their specific protein domains and recruit effectors to regulate the associated gene transcription. These writers/erasers-readers-effectors also participate in the regulation of transcription elongation directly or indirectly.

For the writers, DOT1L is a methyltransferase that can produce mono, di-, and trimethylation on histone H3K79. Dotl1 has a crucial function in embryonic development since Dot1L-null mouse embryos die around 10.5 days (Jones et al., 2008). The distribution of histone H3K79 methylation is highly correlated with effective transcription elongation (Veloso et al., 2014). Although depletion of DOT1L has negligible effects on ESC self-renewal, DOT1L can cooperation with SEC to regulate transcription elongation in an H3K79 methylation-independent manner (Cao et al., 2020).

For the readers, Npac, which can recognize histone H3K36me3 in active gene bodies, positively regulates transcription elongation at key pluripotent genes *Nanog* and *Rif1* to maintain the identity of ESCs (Yu S et al., 2021). Importantly, Npac directly interacts with p-TEFb and Ser5-phosphorylated Pol II (Pol II Ser5P). Depleting Npac impairs the ESC pluripotency by disrupting transcriptional elongation of the pluripotency genes, suggesting that control of transcriptional elongation is crucial for pluripotency maintenance in ESCs.

BRD4 is another reader which possesses two bromodomains and one BET domain. BRD4 can bind to acetylated histone proteins (Taniguchi, 2016). As one of the main partners of p-TEFb, BRD4 exerts a fundamental function in the regulation of transcription elongation (Jang et al., 2005). In ESCs, BRD4 controls transcriptional elongation of super-enhancer (SE)-associated pluripotency genes by recruiting the Mediator and a p-TEFb subunit CDK9 to maintain pluripotency. BRD4 depletion results in differentiation into the neuroectodermal lineage owing to significantly reduced transcription elongation of SE-associated pluripotency genes, such as *POU5F1* and *PRDM14* (Di Micco et al., 2014). Detailed analysis of transcription at single-molecule and single-gene level by further revealed that BRD4 controls Pol II pause release at *Pou5f1* by “Seeding” rather than ‘‘Scaffolding’’ CDK9 clustering, in which BRD4 residence time is much shorter than CDK9 at *Pou5f1* locus (Li et al., 2019). Thus, specific regulation of transcriptional elongation of stem cell genes relies on BRD4-dependent binding of Mediator and CDK9 to SEs to sustain ESC identity.

For the erasers, KDM5B (H3K4me3 demethylase) plays a critical role in controlling self-renewal and pluripotency through regulating transcription elongation and alternative splicing in ESCs (Albert et al., 2013). Histone H3K4me3 is a mark of active transcription and facilities Pol II recruitment to promoter regions. Disruption of the level of H3K4me3 leads to reduce the promoter-proximal pausing Pol II occupancy and elongation rate, accompanied by varied AS. Constantly, depletion of KDM5B leads to re-distribution of histone H3K4 methylation and reduces Pol II Ser5P level in ESCs. Besides, KDM5B is highly enriched at alternatively spliced exons and depletion of KDM5B leads to alternatively spliced exons (He and Kidder, 2017).

Heterochromatin protein 1 (HP1) is known as epigenetic ‘reader’ of a gene repression mark, histone H3K9 methylation. HP1γ (but not HP1α/β) interacts with pluripotency factors (OCT4 and DPPA4) and transcription elongation-associated factors such as FACT complex in ESCs, suggesting that HP1 role in controlling the ESCs identity (Zaidan et al., 2018). Interestingly, HP1γ is predominantly co-localized with H3K36me3 and H3K79me2 rather than H3K9me3 in genic regions of ESCs. In addition, depletion of HP1γ impairs ESC self-renewal, reduces H3K36me3 enrichment and elongation rate at large number of multi-exonic genes that are controlled by c-MYC. Mechanically, HP1γ can interact with NSD1, an H3K36 methyltransferase to maintain the level of H3K36me3 on active transcribes gene body regions (Zaidan and Sridharan, 2020).

### 4.2 Transcription factors regulate the process of transcription elongation

Transcription factors (TFs) can interact with specific DNA sequence and are crucial for the determination and maintenance of the cell’s phenotype (Takahashi and Yamanaka, 2006). General TFs include TFIIA, TFIIB, TFIID, TFIIE, TFIIF, and TFIIH are necessary for transcription initiation (Juven-Gershon and Kadonaga, 2010; Michel and Cramer, 2013). The regulatory TFs can recognize specific DNA sequences (TF binding sites) to control gene expression in a temporal and spatial manner. It is noteworthy that regulatory TFs participate in the process of transcription elongation to determine the cell identity.

In ESCs, c-Myc, is a key target of the LIF/STAT3 signaling pathway to maintain self-renewal and pluripotency. Inhibition of c-Myc antagonizes self-renewal and promotes differentiation while activating stable c-Myc expression renders self-renewal and maintenance of pluripotency even though without LIF (Cartwright et al., 2005). Unlike transcription factor OCT4 which maintains the levels of both promoter-proximal Pol II and elongating Pol II at its target genes, c-Myc only affects the levels of elongating Pol II (Rahl and Young, 2014). Moreover, c-Myc can recruit p-TEFb through directly interacting with cyclin T at its target gene to stimulate productive transcription elongation and loss of c-Myc reduces Pol II density in c-Myc target genes (Rahl et al., 2010).

In other cellular contexts, AIRE, can recruit cyclin T1 of p-TEFb to the site of Pol II pausing regions to induce the production of full-length pre-mRNA in epithelial cells (Oven et al., 2007). NF-κB can directly interact with p-TEFb through binding to the cyclin T1 (Barboric et al., 2001; Nowak et al., 2008). Ikaros, a transcription factor to regulate the expression of the human β globin in hematopoietic cells (Keys et al., 2008), can not only facilitate the assembly of the preinitiation complex but also supports the conversion of Pol II into a productive elongation form by directly interacting with the CDK9 protein kinase (Bottardi et al., 2011). Interestingly, Runx1 can inactivate p-TEFb through binding to cyclin T1 in T cells (Jiang et al., 2005). Recently, NDF (also named as NPAC/GLYR1), a bilaterian nucleosome-destabilizing factor, was identified as a conserved Pol II transcription factor that stimulates elongation (Fei et al., 2022). It will of great interest to examine whether there are more TFs are involved in transcription elongation and how they play their regulatory roles in ESC transcription.

## Perspectives

As discussed above, regulation of transcription elongation has important roles in ESC identity and differentiation. However, there are still some unknown questions to be addressed: 1) Are there any novel players in transcription elongation in ESCs? 2) How do transcription factors, epigenetic modifiers and other co-factors nicely cooperate to control transcription elongation? 3) How do dysfunctions of transcription elongation affect cellular functions and how to rescue the defects caused? 4) How is transcription elongation coupled with RNA splicing? Hence, more investigations are required to provide more insights into the regulatory mechanism of transcription elongation. Besides, more advanced strategies to study transcription elongation are expected. Non-etheless, the emerging auxin-inducible degradation system which can rapidly degrade proteins and the development of small molecular inhibitors which can specifically target specific protein domains allow researchers to study transcription elongation more efficiently. In addition, the use of high-resolution microscopy and high-throughput sequencing will provide unprecedented opportunities to fully understand transcription elongation in ESCs.
